# Molecular separation of ions from aqueous solutions using modified nanocomposites

**DOI:** 10.1038/s41598-021-89371-5

**Published:** 2021-06-30

**Authors:** Hamed Ghaforinejad, Azam Marjani, Hossein Mazaheri, Ali Hassani Joshaghani

**Affiliations:** 1 Department of Chemical Engineering, Arak Branch, Islamic Azad University, Arak, Iran; 2 Department of Chemistry, Arak Branch, Islamic Azad University, Arak, Iran

**Keywords:** Climate sciences, Environmental sciences

## Abstract

Herein, two novel porous polymer matrix nanocomposites were synthesized and used as adsorbents for heavy metal uptake. Methacrylate-modified large mesoporous silica FDU-12 was incorporated in poly(methyl methacrylate) matrix through an in-situ polymerization approach. For another, amine-modified FDU-12 was composited with Nylon 6,6 via a facile solution blending protocol. Various characterization techniques including small-angle X-ray scattering, FTIR spectroscopy, field emission-scanning electron microscopy, transmission electron microscopy, porosimetry, and thermogravimetric analysis have been applied to investigate the physical and chemical properties of the prepared materials. The adsorption of Pb(II) onto the synthesized nanocomposites was studied in a batch system. After study the effect of solution pH, adsorbent amount, contact time, and initial concentration of metal ion on the adsorption process, kinetic studies were also conducted. For both adsorbents, the Langmuir and pseudo-second-order models were found to be the best fit to predict isotherm and kinetics of adsorption. Based on the Langmuir model, maximum adsorption capacities of 105.3 and 109.9 mg g^−1^ were obtained for methacrylate-modified FDU-12/poly(methyl methacrylate) and amine-modified FDU-12/Nylon 6,6, respectively.

## Introduction

As an important category of materials with increasing applications in various branches of science, porous materials have been applied for catalysis, energy storage, drug delivery, adsorption, and sample preparation^1–13^. Recently, adsorption has attracted much attention in separation and removal of water pollutants with high efficiency^14–20^. As a subgroup of porous materials for adsorption of pollutants, ordered mesoporous materials exhibited highly ordered structure, large pore volume, high surface area, and functionalizable surface^21–28^. These characteristics make ordered mesoporous materials suitable for a broad range of applications, such as adsorption^29–31^. Various silica-based mesoporous materials such as SBA, MCM, KIT, and FDU families have been introduced during the last years. Among them, those with three-dimensional architecture (e.g. SBA-16, MCM-48, KIT-6, FDU-12) provided a suitable mass transfer for the diffusion of guest molecules^32^. In this regard, FDU-12 is a member of mesoporous silica materials with a highly ordered structure, high specific surface area, adjustable pore size, large pore diameter, and superior 3D channel^33,34^ which can be synthesized with different pore size distributions as reported in the literature^35,36^.

As an important application of mesoporous materials, their use in the nanocomposite industry as nanofiller has attracted a lot of attention owing to their unique properties. Nanocomposite materials provided a higher surface area to volume ratio in comparison to the neat polymer^37^. The use of mesoporous materials as filler in polymeric nanocomposites gathering the flexibility of organic polymers and the advantages of mesoporous materials such as high thermal and mechanical stability, and high surface area. Various organic polymers have been applied for the preparation of nanocomposites containing mesoporous materials. Among them, poly(methyl methacrylate) (PMMA), and Nylon have been widely utilized due to their unique advantages. PMMA is a thermoplastic, transparent, and rigid polymer with good chemical resistance, good flexibility, low density, and low cost^38^. Nylon is a polar, electron-rich synthetic polyamide with a porous structure^39^ composed of microfibrils that are interconnected forming a three-dimensional network. Among commercial types of Nylon, Nylon 6 and Nylon 6,6 continue to be the most popular types*.*

The incorporation of nanofiller (e.g. silica-based mesoporous materials) into the organic polymer matrix can be attained by various methods such as melt blending, solution blending, in-situ polymerization, etc.^40,41^. Each of these methods has its advantages and disadvantages which were discussed in detail in the literature^40,41^. Briefly, in the melt blending technique, the polymer is melted and combined with the nanofiller to obtain polymer nanocomposite. In the case of solution blending, the nanofiller and organic polymer are dispersed in suitable solvents and then mixed by agitation (magnetic stirring, sonication, etc.) followed by evaporation of the solvent and composite film casting. In the in-situ polymerization technique, the nanofiller is dispersed into the monomer solution followed by polymerization of the mixture by the addition of a suitable catalyst (polymerization initiator).

To expand the applicability of mesoporous silica materials (e.g., FDU-12) and applying them as nanofiller in the polymer nanocomposites, their hydrophobicity and surface characteristics should be changed to enhance the compatibility of the nanofiller and polymer matrix. In this case, the incorporation of organic functional groups onto the ordered structure of the mesoporous silica materials seems to be a good choice. Surface functionalization of mesoporous silica materials with a suitable functional group could help linking up the filler with the polymer matrix through functional groups^37,42–46^. On the other hand, more effective dispersion and penetration occur. Due to the presence of a large number of hydroxyl groups on the surface of mesoporous silica materials, surface functionalization with a variety of organic moieties seems to be relatively easy^47^. In this case, there are few reports regarding the functionalization of mesoporous silica FDU-12^48–52^.

Among the diverse applications of polymer-based nanocomposites, their use in adsorption technique has received lots of attention. Due to their good performance, structural diversity, low toxicity, and low cost, polymer matrix nanocomposites are known as promising adsorbents for decontamination of polluted waters via adsorption technique^37^. In the present paper, two novel modified mesoporous silica FDU-12 materials were synthesized for incorporation into PMMA and Nylon 6,6 polymers through in-situ polymerization and solution blending techniques. Surface modification of the prepared mesoporous silica FDU-12 was performed using silane coupling agents of 3-(triethoxysilyl)propyl methacrylate and *N*^1^-(3-trimethoxysilylpropyl)diethylenetriamine. The prepared nanocomposites were applied as new adsorbents for Pb(II) uptake from aqueous solutions.

## Results and discussion

### Characterization

Various characterization techniques including SAXS, FT-IR, FE-SEM, TEM, N_2_ adsorption/desorption, and TGA were applied to examine the chemical structure and morphology of the prepared materials.

**Figure 1:**
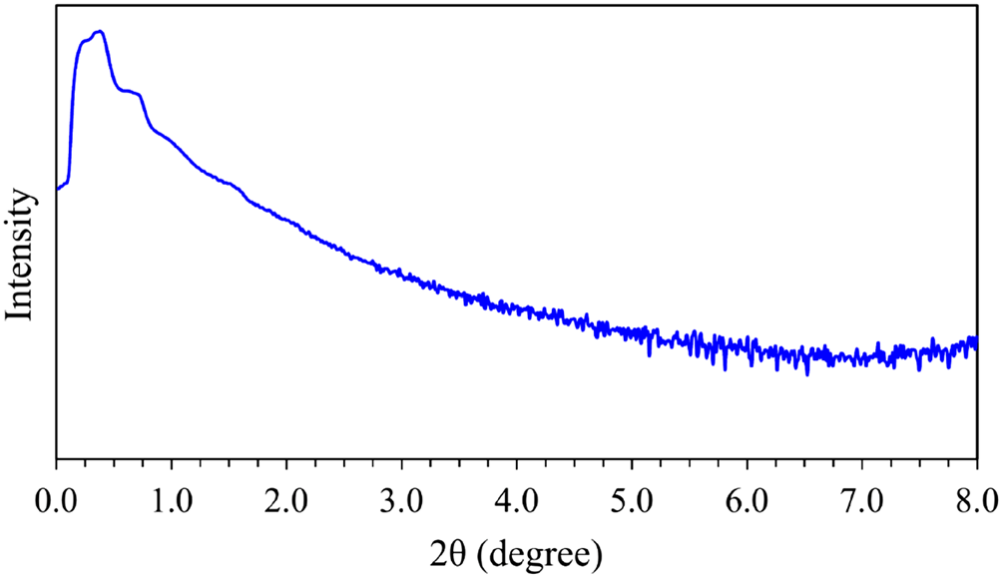
The SAXS pattern of the prepared pristine FDU-12.

The SAXS pattern of the pristine mesoporous silica FDU-12 is shown in Fig. 1. As can be seen, the prepared sample exhibits some characteristic peaks especially at 2θ below 0.5. This pattern is similar to that of FDU-12 reported in the literature indicating the successful synthesis of ordered FDU-12 mesoporous silica^36,47,48,53,54^.

**Figure 2:**
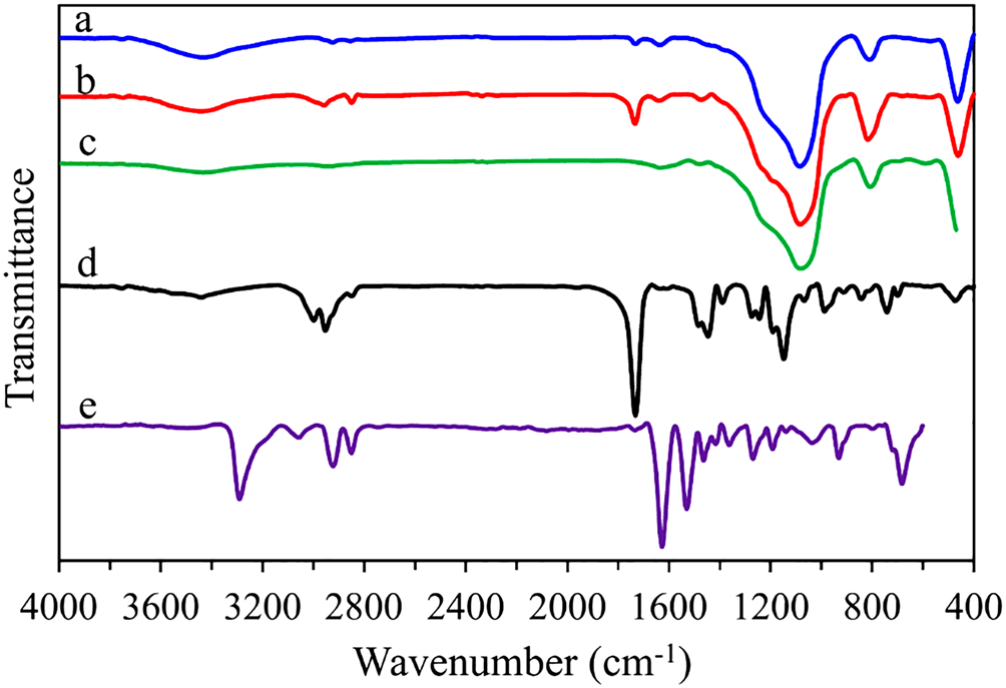
The FT-IR spectra of **(a)** pristine FDU-12, **(b)** FDU-12-methacrylate, **(c)** FDU-12-triamine, **(d)** FDU-12-methacrylate/PMMA, and **(e)** FDU-12-triamine/NY6,6.

Figure 2 shows the FT-IR spectra of pristine FDU-12, FDU-12-methacrylate, FDU-12-triamine, FDU-12-methacrylate/PMMA, and FDU-12-triamine/NY6,6. In the pristine FDU-12 spectrum (Fig. 2a), the bands at 463, 810, and 1078 cm^−1^ are attributed to the Si–O–Si vibrations which are in agreement with previous reports^33,34,55^. The broadband around 3435 cm^−1^ corresponds to O–H stretching of the surface silanol groups and physically adsorbed water molecules. Also, the band located at 1633 cm^−1^ is attributed to the bending mode of O–H. In the case of FDU-12-methacrylate and FDU-12-triamine (Fig. 2b,c), in addition to the bands observed for pristine FDU-12, new bands (related to the silane coupling agent) appeared. The bands located at 2957 and 2849 cm^−1^ correspond to the C–H stretching vibrations and the peak at 1471 cm^−1^ is corresponds to the bending vibration of the C–H of the organic part of the silane coupling agent. For FDU-12-methacrylate, the band at 1733 cm^−1^ is characteristic of the C=O bond. In the case of FDU-12-triamine, the broadband at 3200–3600 cm^−1^ revealed the presence of –NH_2_ and –OH groups on the surface of the material. The FTIR spectra of FDU-12-methacrylate and FDU-12-triamine indicated successful surface modification of the pristine FDU-12 with the applied silane coupling agents. The FT-IR spectrum of FDU-12-methacrylate/PMMA material (Fig. 2d) shows C–H asymmetric stretching bands located at 2998 and 2952 cm^−1^ for CH_3_ and CH_2_ groups, respectively. A peak that appeared at 2849 cm^−1^ is related to the C–H symmetric stretching in the CH_3_ group. The characteristic band at 1731 cm^−1^ corresponds to the C=O bond. Other characteristic bands related to PMMA also appear in the spectrum of FDU-12-methacrylate/PMMA correspond to different modes of CH_2_ and CH_3_ vibrational modes^38,56^. In the case of FDU-12-triamine/NY6,6 (Fig. 2e), the characteristic peaks at 3290, 2849 & 2921 cm^−1^ correspond to the stretching vibration of the N–H bond and C-H stretching vibrations of the NY6,6. The peaks centered at 1627 and 1530 cm^−1^ are assigned to the stretching vibration of carbonyl groups and N–H bending vibration. Other characteristic bands related to NY6,6 also appeared in the spectrum of FDU-12-triamine/NY6,6 as reported in the literature^57,58^.

**Figure 3:**
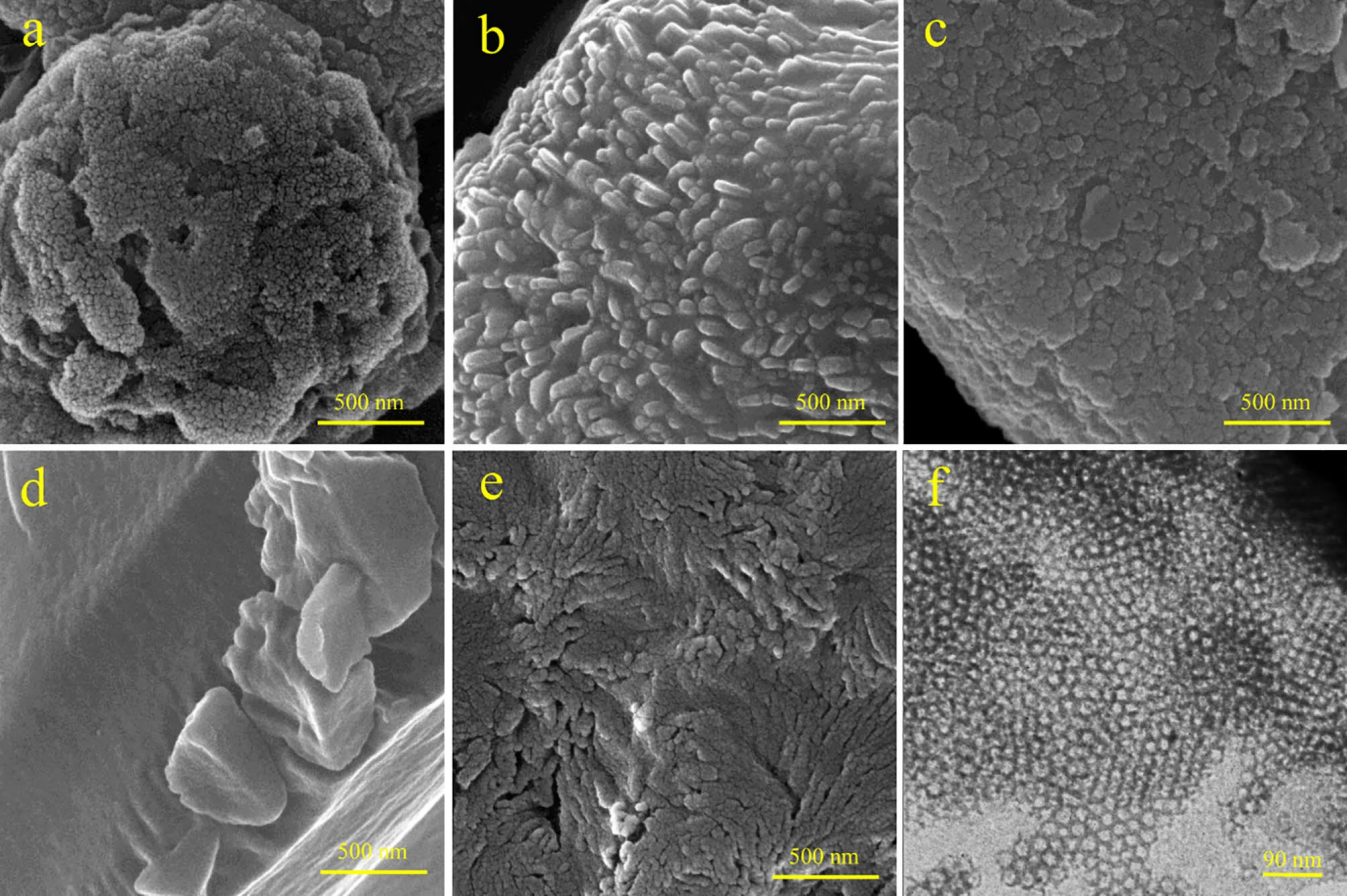
The FE-SEM images of **(a)** pristine FDU-12, **(b)** FDU-12-methacrylate, **(c)** FDU-12-triamine, **(d)** FDU-12-methacrylate/PMMA, **(e)** FDU-12-triamine/NY6,6, and **(f)** TEM image of pristine FDU-12.

To study the morphological characteristics of the prepared materials, FE-SEM, and TEM techniques were applied. The FE-SEM images of the pristine FDU-12, FDU-12-methacrylate, FDU-12-triamine, FDU-12-methacrylate/PMMA, and FDU-12-triamine/NY6,6 are shown in Fig. 3a–e. As can be seen, Fig. 3a shows the porous structure of pristine FDU-12. After surface modification with silane coupling agents of 3-(triethoxysilyl)propyl methacrylate (Fig. 3b) and *N*^1^-(3-trimethoxysilylpropyl)diethylenetriamine (Fig. 3c), a slight change in the surface morphology has occurred and the surface of FDU-12 turned smoother. This is due to the silane coupling agents grafting on the surface of FDU-12. In the case of FDU-12-methacrylate/PMMA (Fig. 3d), a monolithic and relatively smooth surface morphology was observed while the FDU-12-triamine/NY6,6 (Fig. 3e) showed more porous structure which is a characteristic of Ny6,6 morphology. The TEM image of the synthesized pristine FDU-12 is also shown in Fig. 3f. The TEM image clearly demonstrated the well-ordered mesopore structure with large cage units of FDU-12 as reported in the literature^53,54,59^.

**Figure 4:**
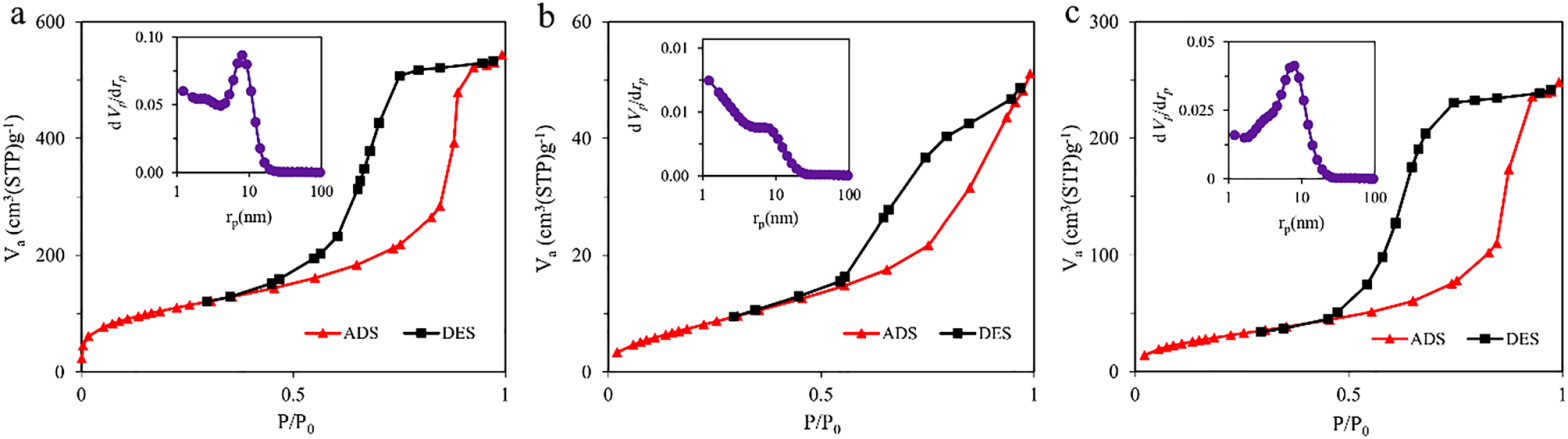
N_2_ adsorption/desorption isotherms of **(a)** pristine FDU-12, **(b)** FDU-12-methacrylate, and **(c)** FDU-12-triamine. The BJH pore size distribution curves of the samples are also shown.

The N_2_ adsorption/desorption measurements of the pristine FDU-12, FDU-12-methacrylate, and FDU-12-triamine are shown in Fig. 4. The three samples showed type IV isotherms with H2-type hysteresis loops according to the IUPAC classification. The BET surface areas of the samples were obtained 376, 29, and 115 m^2^ g^-1^ for FDU-12, FDU-12-methacrylate, and FDU-12-triamine, respectively. The decrease in the BET surface area may be due to surface modification with silane coupling agents which is in agreement with the results obtained by FE-SEM analysis. The mean pore diameters according to the BJH model are also shown in Fig. 4. Mean pore diameters of 9.2, 1.2, and 6.9 nm were calculated for pristine FDU-12, FDU-12-methacrylate, and FDU-12-triamine, respectively. As data showed, after surface modification, the textural parameters of the pristine FDU-12 have reduced as it was anticipatable. We applied a post-modification strategy for the surface modification of FDU-12. This strategy suffers from the pore-blocking problem which means the pores of the porous material are blocked by silane coupling agents which results in a decrease in the accessible surface area. We propose that pore-blocking happens more intensely in the case of 3-(triethoxysilyl)propyl methacrylate rather than N^1^-(3-trimethoxysilylpropyl)diethylenetriamine due to more reactivity of methacrylate-based coupling agent. As a result, FDU-12- methacrylate shows a lower surface area than FDU-12- triamine.

**Figure 5:**
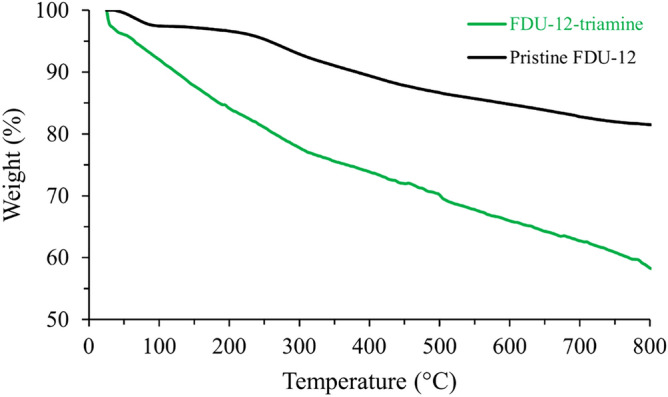
TGA curves of pristine FDU-12 and FDU-12-triamine.

To study the thermal stability of the prepared materials, thermogravimetric analysis was performed for pristine FDU-12 and FDU-12-triamine. The results are illustrated in Fig. 5. A weight loss of 18% was observed for the pristine FDU-12 sample. In the case of FDU-12-triamine, a continuous weight loss was observed. A relatively large loss (42%) was observed for FDU-12-triamine. The difference between the final weight loss of the two samples was related to modifier loaded on the surface of pristine FDU-12.

### Adsorption studies

#### The effect of pH

**Figure 6:**
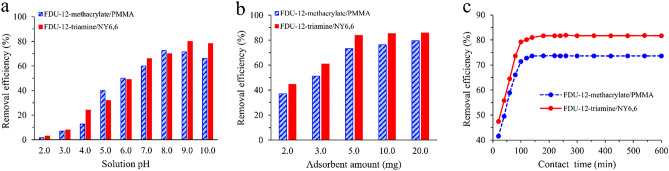
The effect of **(a)** pH, **(b)** adsorbent amount, and **(c)** contact time on the adsorption process.

The effect of pH on the adsorption of Pb(II) onto the prepared FDU-12-methacrylate/PMMA and FDU-12-triamine/NY6,6 adsorbents was studied in the range of 2.0–10.0. In this step, 12 mL of an aqueous standard solution of Pb(II) (50 mg L^-1^) with 5.0 mg of each adsorbent was considered. All the adsorption experiments were performed at 298 K for 12 h (180 rpm). As can be seen in Fig. 6a, for FDU-12-methacrylate/PMMA, the removal efficiency was enhanced with increasing solution pH up to 8.0 and then a slight decrease in the removal efficiency was observed for greater pHs. A similar trend was observed for FDU-12-triamine/NY6,6 with maximum removal efficiency at pH 9.0. In acidic mediums, the competition between H^+^ and Pb(II) ions in binding to the active sites of the adsorbent caused a decrease in removal efficiency. Accordingly, pH 8.0 and pH 9.0 were selected for further experiments using FDU-12-methacrylate/PMMA and FDU-12-triamine/NY6,6, respectively.

#### The effect of the adsorbent amount

The effect of adsorbent amount on the adsorption of Pb(II) was investigated by studying various adsorbent amount in the range of 2.0–20.0 mg. In these experiments, 12 mL of standard solution of Pb(II) with the concentration of 50 mg L^-1^ (pH = 8.0 for FDU-12-methacrylate/PMMA and pH = 9.0 for FDU-12-triamine/NY6,6) were used. The adsorption experiments were performed at 298 K for 12 h (180 rpm). As data in Fig. 6b shows, the removal efficiency was increased with enhancing adsorbent amount from 2.0 to 5.0 mg for both FDU-12-methacrylate/PMMA and FDU-12-triamine/NY6,6. In the case of both adsorbents, no important increase in the removal efficiency was observed for higher adsorbent amounts. Based on the results obtained from the experiments, an amount of 5.0 mg of each of the adsorbents was selected for further experiments.

#### The effect of contact time

To study the effect of contact time on adsorption efficiency, various contact times between 20 and 600 min were tested. In this step, 12 mL of a standard solution of Pb(II) at the concentration level of 50 mg L^-1^ (pH = 8.0 for FDU-12-methacrylate/PMMA and pH = 9.0 for FDU-12-triamine/NY6,6) and 5.0 mg of each sorbent was used. Adsorptions were performed at 298 K and 180 rpm. As can be seen in Fig. 6c, the removal efficiencies using both FDU-12-methacrylate/PMMA and FDU-12-triamine/NY6,6 were sharply increased with increasing contact time from 20 to 100 min. A slight enhancement in the removal efficiencies was also observed from 100 to 140 min. Longer contact times did not affect the removal efficiency in the case of both adsorbents. This relatively fast adsorption was mainly due to the high accessible sites on the surface of FDU-12-methacrylate/PMMA and FDU-12-triamine/NY6,6 adsorbents. In conclusion, the contact time was set to 140 min for further experiments to ensure that equilibrium is reached.

#### Adsorption kinetic studies

Four kinetic models including pseudo-first-order (PFO), pseudo-second-order (PSO), Elovich, and intraparticle diffusion (IPD) were used to conduct kinetic studies. The equation of the applied kinetic models are as follows:

1 $$\mathit{log}\left({q}_{e}-{q}_{t}\right)=\mathit{log}{q}_{e}-\frac{{k}_{1}}{2.303}t$$

2 $$\frac{t}{{q}_{t}}=\frac{1}{{k}_{2}\times {q}_{e}^{2}}+\frac{1}{{q}_{e}}t$$

3 $${q}_{t}=\frac{\mathit{ln} \left(\alpha \beta \right)}{\beta }+\frac{\mathit{ln}t}{\beta }$$

4 $${q}_{t}={k}_{dif}({t)}^{0.5}+C$$

**Figure 7:**
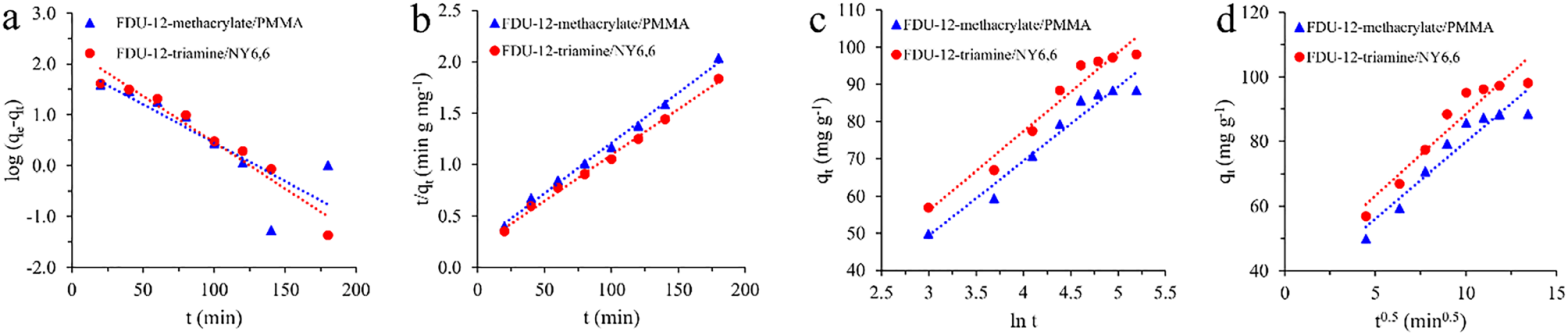
The kinetic adsorption models of **(a)** pseudo-first-order, **(b)** pseudo-second-order, **(c)** Elovich, and **(d)** intra-particle diffusion for the adsorption of Pb(II) onto the FDU-12-methacrylate/PMMA and FDU-12-triamine/NY6,6.

Also, *q*_*e*_, *q*_*t*_, *k*_*1*_, *k*_*2*_, *α* & *β*, and *k*_*dif*_ are the adsorption capacity at equilibrium (mg g^-1^), the adsorption capacity at time t (mg g^-1^), PFO rate constant (min^-1^), PSO rate constant (g mg^-1^ min^-1^), Elovich constants (mg g^-1^ min^-1^ & g mg^-1^), IPD rate constant (mg g^-1^ min^-0.5^), and a constant (mg g^-1^), respectively. The results of the fitting are illustrated in Fig. 7 and Table 1. The data showed the PSO kinetic model provided the best fit in the case of both adsorbents. The R^2^ values obtained by PSO kinetic model were obtained 0.9952 and 0.9955 for FDU-12-methacrylate/PMMA and FDU-12-triamine/NY6,6, respectively. Accordingly, the chemical adsorption process can be described with the PSO model. On the other hand, the presence of a large number of hydroxyl, amine, and carbonyl groups on the surface of FDU-12-methacrylate/PMMA and FDU-12-triamine/NY6,6 might be involved in the process.

**Table 1:** Parameters obtained by kinetic models for the adsorption of Pb(II) onto the prepared adsorbents.

Model	Adsorbent	R^2^	Parameters^a^	
PFO	FDU-12-methacrylate/PMMA	0.7005	*k*_*1*_ = 0.00012	*q*_*e*_ = 66.7
	FDU-12-triamine/NY6,6	0.9497	*k*_*1*_ = 0.00015	*q*_*e*_ = 54.9
PSO	FDU-12-methacrylate/PMMA	0.9952	*k*_*2*_ = 0.00042	*q*_*e*_ = 102.0
	FDU-12-triamine/NY6,6	0.9955	*k*_*2*_ = 0.00039	*q*_*e*_ = 112.4
Elovich	FDU-12-methacrylate/PMMA	0.9571	*α* = 11.75982	*β* = 0.05000
	FDU-12-triamine/NY6,6	0.9553	*α* = 14.79877	*β* = 0.04702
IPD	FDU-12-methacrylate/PMMA	0.9042	*k*_*dif*_ = 4.74950	*C* = 32.36240
	FDU-12-triamine/NY6,6	0.9070	*k*_*dif*_ = 5.06240	*C* = 37.87973

^a^The units are as same as mentioned in the section “Adsorption kinetic studies”.

#### The effect of Pb(II) initial concentration and adsorption isotherm

**Figure 8:**
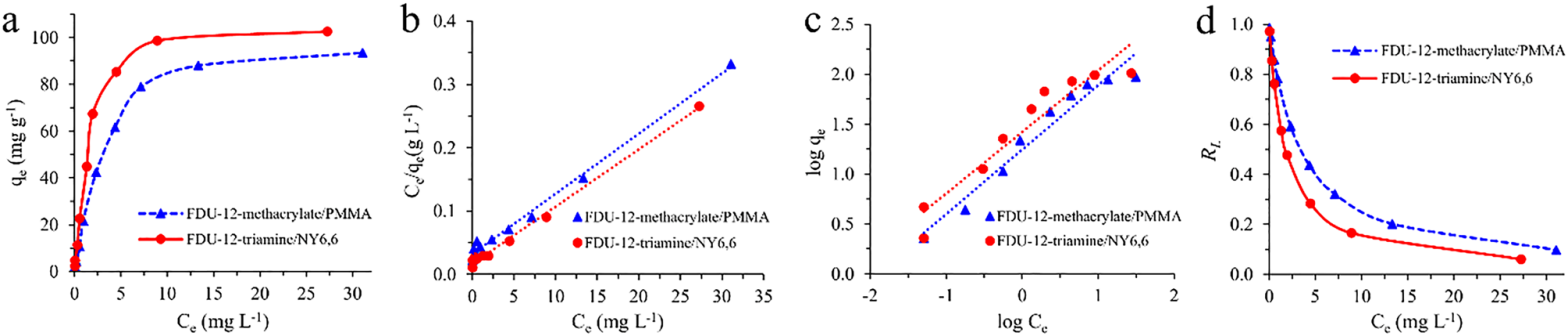
**(a)** The equilibrium isotherm and the isotherm models of **(b)** Langmuir, **(c)** Freundlich, and **(d)** the calculated values of *R*_*L*_ for the adsorption of Pb(II) onto the FDU-12-methacrylate/PMMA and FDU-12-triamine/NY6,6.

The effect of the initial concentration of Pb(II) on the adsorption process was studied in the range of 1.0 to 70.0 mg L^-1^ using FDU-12-methacrylate/PMMA and FDU-12-triamine/NY6,6 as adsorbents (Fig. 8a). In these experiments, 12 mL of the standard solution of Pb(II) (pH  8.0 for FDU-12-methacrylate/PMMA and pH  9.0 for FDU-12-triamine/NY6,6) with 5.0 mg of each adsorbent were used and the experiments were conducted at 298 K and 180 rpm. The contact time was set to 140 min for both adsorbents. To study the adsorption isotherm, two isotherm models including Langmuir and Freundlich were applied. The linear form of the used isotherm models is expressed as follows:

5 $$\frac{{C}_{e}}{{q}_{e}}=\frac{1}{{q}_{max}\times {k}_{L}}+\frac{{C}_{e}}{{q}_{max}}$$

6 $$\mathit{log}{q}_{e}=\frac{1}{n}\mathit{log}{C}_{e}+\mathit{log}{k}_{F}$$

In the equations, *C*_*e*_ is the concentration of Pb(II) at equilibrium (mg L^-1^), *q*_*e*_ is the adsorption capacity at equilibrium (mg g^-1^), *q*_*max*_ is the maximum adsorption capacity of adsorbent (mg g^-1^), *k*_*L*_ is the Langmuir constant (L mg^-1^), and *n* and *k*_*F*_ ((mg g^-1^) (L mg^-1^)^1/n^) are the Freundlich isotherm constants. The adsorption isotherms and calculated parameters are shown in Fig. 8b–d and Table 2, respectively. As data in Table 2 show, the Langmuir model exhibited the best fit to the experimental data considering the R^2^ value (0.9926 and 0.9955 for FDU-12-methacrylate/PMMA and FDU-12-triamine/NY6,6, respectively). The maximum adsorption capacities of FDU-12-methacrylate/PMMA and FDU-12-triamine/NY6,6 obtained by the Langmuir model were found to be 105.3 and 109.9 mg g^−1^, respectively. In the Langmuir adsorption isotherm model, it is assumed that the energy of adsorption is constant and there is a fixed number of identical adsorption sites on the adsorbent surface. In the next step, the *R*_*L*_ value was also calculated to predict adsorption performance. It can be defined as follows:

**Table 2:** Parameters obtained by isotherm models for the adsorption of Pb(II) onto the prepared adsorbents.

Model	Adsorbent	R^2^	Parameters^a^	
Langmuir	FDU-12-methacrylate/PMMA	0.9926	*k*_*L*_ = 0.296	*q*_*max*_ = 105.3
	FDU-12-triamine/NY6,6	0.9955	*k*_*L*_ = 0.562	*q*_*max*_ = 109.9
Freundlich	FDU-12-methacrylate/PMMA	0.9521	*k*_*F*_ = 17.579	*n* = 1.548
	FDU-12-triamine/NY6,6	0.9134	*k*_*F*_ = 26.430	*n* = 1.621

^a^The units are as same as mentioned in the section “The effect of Pb(II) initial concentration and adsorption isotherm”.

7 $${R}_{L}=\frac{1}{{1+(C}_{e}{k}_{L})}$$

The calculated *R*_*L*_ values are shown in Fig. 8d. In the case of both adsorbents, the *R*_*L*_ values were obtained between 0 and 1 which represents favorable adsorption of Pb(II) onto the FDU-12-methacrylate/PMMA and FDU-12-triamine/NY6,6.

Table 3 shows data from the previous studies for the removal of Pb(II). The FDU-12-methacrylate/PMMA and FDU-12-triamine/NY6,6 adsorbents exhibited promising Pb(II) adsorption capacity when compared to other adsorbents.

**Table 3:** Comparison of the adsorption capacity of the prepared adsorbents toward Pb(II) ions with other adsorbents.

Adsorbent	*q*_*max*_ (mg g^-1^)	pH	References
Oxidized multiwalled carbon nanotubes/polypyrrole composite	26.32	6.0	^60^
SBA-15-supported Pb(II) imprinted polymer	42.55	6.0	^61^
Oil palm bio-waste/multiwalled carbon nanotubes reinforced PVA hydrogel	30.03	7.0	^62^
Triamino-functionalized KCC-1/chitosan-oleic acid nanocomposites	168	9.0	^63^
Fe_3_O_4_/cyclodextrin polymer nanocomposite	64.5	5.5	^64^
FDU-12-methacrylate/PMMA	105.3	8.0	This work
FDU-12-triamine/NY6,6	109.9	9.0	This work

#### Recyclability of the adsorbents

To study the recyclability and reusability of the prepared adsorbents, at first, 12 mL of the standard solution of Pb(II) (50 mg L^-1^, pH = 8.0 for FDU-12-methacrylate/PMMA and pH = 9.0 for FDU-12-triamine/NY6,6) with 5.0 mg of each adsorbent were used and the experiments were conducted at 298 K and 180 rpm. The contact time was set to 140 min for both adsorbents. After equilibrium, the adsorbents were isolated from the solution by centrifugation. Desorption was performed using 100 mL of 0.1 mol L^-1^ of HCl solution for 120 min. Then, the adsorbents were rinsed several times with pure water, dried, and used for further experiments. In the case of both adsorbents, the adsorption capacity remained approximately constant for the three cycles and then decreased 25–30% after the fourth cycle. This decrease was probably attributed to the weight loss of adsorbent in the consecutive adsorption/desorption cycles.

##### Selectivity tests

**Figure 9:**
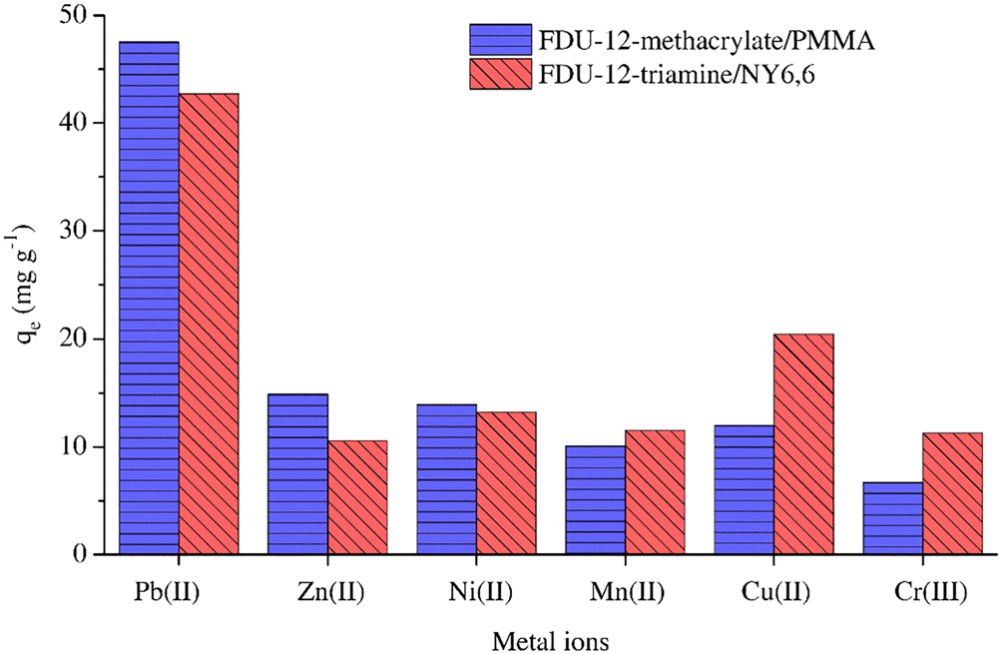
Selectivity of FDU-12-methacrylate/PMMA and FDU-12-triamine/NY6,6 in the presence of coexisting metal ions.

5.0 mg adsorbent (FDU-12-methacrylate/PMMA or FDU-12-triamine/NY6,6) was added to an aqueous solution (12 mL) containing 50 mg L^–1^ of Pb(II), Zn(II), Ni(II), Mn(II), Cu(II), and Cr(III) diluted from the commercial stock solution and further analyzed by inductively coupled plasma optical emission spectrometry (ICP-OES). The mixture was then shaken at 180 rpm and 298 K for 140 min. The adsorbent was separated from the mixture and the remain concentration of above-mentioned heavy metals in the solution was analyzed by ICP-OES. As shown in Fig. 9, both adsorbents exhibited acceptable selectivity to adsorb Pb(II) in the presence of all other competitive ions. These results demonstrate that these adsorbents can be beneficial for practical adsorption application, i.e., the adsorption of Pb(II) from aqueous media in the presence of other coexisting meal ions.

## Conclusions

To expand the applicability of mesoporous silica materials and applying them as nanofiller in the polymer matrix nanocomposites, in this study, at first, a three-dimensional large mesoporous silica (FDU-12) was synthesized and then modified with 3-(triethoxysilyl)propyl methacrylate and *N*^1^-(3-trimethoxysilylpropyl)diethylenetriamine. This step was performed to enhance the compatibility of the nanofiller and polymer matrix by changing the hydrophobicity and surface characteristics of the prepared mesoporous silica. After surface modification of the mesoporous silica material, they were used as nanofiller for the preparation of PMMA- and NY6,6-based nanocomposites. The newly nanocomposites were prepared through two synthesis procedures. The FDU-12-methacrylate/PMMA was fabricated using the in-situ polymerization technique while the FDU-12-triamine/NY6,6 was prepared via a simple and fast solution blending technique. Due to the abundant active sites on the surface of the adsorbents, relatively high surface area, and various types of interactions, the prepared adsorbents showed good performance toward Pb(II) uptake from aqueous media. The studies on the affecting parameters on the adsorption process revealed that the best performance was obtained at a sample solution pH of 8.0 and 9.0 (for FDU-12-methacrylate/PMMA and FDU-12-triamine/NY6,6, respectively), adsorbent amount of 5.0 mg, and contact time of 140 min for both nanocomposites. Among the applied kinetic models, the PSO model showed the best fit for the two adsorbents. The isotherm studies were also showed the Langmuir model provided the best matches considering the *R*^*2*^ value. Based on the Langmuir model, the FDU-12-methacrylate/PMMA and FDU-12-triamine/NY6,6 adsorbents provided maximum adsorption capacities of 105.3 and 109.9 mg g^-1^, respectively. The prepared nanocomposites showed good performance and can be considered for Pb(II) removal from aqueous solutions.

## Experimental

### Materials and reagents

Absolute ethanol, toluene, *ortho*-phosphoric acid, hydrochloric acid, formic acid, acetic acid, boric acid, tetraethyl orthosilicate (TEOS), sodium hydroxide, lead(II) nitrate, potassium chloride, and benzoyl peroxide were purchased from Merck (Darmstadt, Germany). Pluronic F-127 (Mw ~ 12,600 Da), 1,3,5-trimethylbenzene (TMB), *N*^1^-(3-trimethoxysilylpropyl)diethylenetriamine, methyl methacrylate, and Nylon 6,6 were obtained from Sigma-Aldrich (St. Louis, MO, USA). 3-(triethoxysilyl)propyl methacrylate was obtained from TCI (Europe). Pure water was prepared via a laboratory-scale water purification system. A stock solution of Pb(II) at the concentration level of 1000 mg L^−1^ was prepared in pure water. The working standard solutions were daily prepared from the stock solution. The Brighton-Robinson buffer system (universal buffer) was used for the preparation of aqueous solutions with different pHs.

### Apparatus and instruments

Small-angle X-ray scattering (SAXS) profiles of the prepared materials were recorded on an S3-MICROpix Hecus X-Ray system (Austria) at 50 kV and 1 mA. A Thermo Nicolet Avatar 330 FTIR spectrometer (USA) was applied for the FTIR analysis of the materials. The spectra were recorded in the range of 4000–400 cm^-1^. The resolution was set to 4 cm^-1^. Porosimetry results were obtained by the nitrogen adsorption/desorption technique at 77 K on a Belsorp-mini II (BEL Japan Inc., Japan) instrument. The adsorption isotherms were measured at 77 K (liquid nitrogen). The FE-SEM and TEM images of the samples were obtained by MIRA3 TESCAN-XMU (Czech Republic) and Philips CM 120 (Eindhoven, The Netherlands) microscopes. 2-propanol was used for sample dispersion before the analysis. Thermogravimetric analysis was performed on a Mettler TGA instrument from 25 to 800 °C. Flame atomic absorption spectroscopy was performed on a 240FS AA instrument (Agilent, USA). Inductively coupled plasma-optical emission spectroscopy was carried out on an Optima 7300 DV inductively coupled plasma optical emission spectrometer (ICP-OES) instrument (PerkinElmer, USA). A MISONIX XL-2000 ultrasonic liquid processor (Raleigh, North Carolina, USA) at a power of 100 W was used to carry out the reactions where needed.

### Synthesis of mesoporous silica FDU-12

At first, pristine mesoporous silica FDU-12 was synthesized according to the method reported in the literature^34,55^ with some modifications. Typically, 3.0 g of Pluronic F-127 was dissolved in 120 mL of HCL (2.0 mol L^-1^). After 20 min, 7.5 g of KCl and 3.0 g of TMB were individually added to the solution and the mixture was stirred at 288 K for 2 h. Then, 12.5 g of TEOS was added to the mixture and the solution was heated at 383 K in an autoclave for 72 h. The obtained solid product was then gathered and washed with pure water and ethanol before oven drying at 343 K for 24 h. Afterward, the material was calcined at 823 K for 5 h.

### Surface modification of FDU-12

To surface modification of the prepared FDU-12, two silane coupling agents of 3-(triethoxysilyl)propyl methacrylate and *N*^1^-(3-trimethoxysilylpropyl)diethylenetriamine were applied utilizing post-synthesis modification according to the method described by Soltani and co-workers^27,37,46^ with a little modification. The synthesized FDU-12 (0.5 g) and silane coupling agent (0.5 mL) were added to 40 mL of dried toluene and the mixture was refluxed at 383 K for 24 h. The obtained product was then isolated with the help of a Büchner funnel, washed with toluene and ethanol, and oven-dried under at 343 K for 24 h. The samples modified with 3-(triethoxysilyl)propyl methacrylate and *N*^1^-(3-trimethoxysilylpropyl)diethylenetriamine were denoted as FDU-12-methacrylate and FDU-12-triamine, respectively.

### Preparation of FDU-12-methacrylate/PMMA nanocomposite through in-situ polymerization approach

For fabrication of FDU-12-methacrylate/PMMA nanocomposite via* in-situ* polymerization approach, 0.15 g of FDU-12-methacrylate was added to 50 mL of dry toluene and the mixture was sonicated for 10 min. Then, 4.85 mL methyl methacrylate monomer was added to the mixture with mechanical stirring to obtain a stable suspension and sonicated for 15 min. Afterward, 1.0 wt% of benzoyl peroxide initiator was added to the mixture and it was refluxed under isothermal conditions for 24 h. After in-situ polymerization, the mixture was sonicated for 15 min and poured into a clean glass Petri dish, and dried at room temperature for 5 h. The FDU-12-methacrylate/PMMA nanocomposite was then obtained in this step.

### Preparation of FDU-12-triamine/NY6,6 nanocomposite

FDU-12-triamine/NY6,6 nanocomposite was fabricated through the solution blending protocol. An amount of 4.85 g of NY6,6 was added to 40 mL of formic acid and the mixture was refluxed under a nitrogen atmosphere for 6 h to obtain a clear solution. Then 3 wt% of FDU-12-triamine was added to 10 mL of formic acid and the mixture was sonicated for 15 min. Then, the FDU-12-triamine mixture was added dropwise to the polymer solution and the mixture was refluxed for 12 h. Afterward, the mixture was sonicated for 1 h and the resultant was cast onto a clean glass Petri dish and dried at room temperature. The FDU-12-triamine/NY6,6 nanocomposite then resulted in this step.

### Adsorption procedure

The adsorption experiments were performed to study the behavior of the prepared materials for the removal of Pb(II). Batch experiments were carried out using 12 mL of an aqueous standard solution of Pb(II) and various adsorbent dosages in 15 mL polyethylene containers. The procedure was performed at 180 rpm and 298 K for a specific period. Then, the adsorbent was separated from the mixture and the remain concentration of Pb(II) in the solution was determined using flame atomic absorption spectroscopy or inductively coupled plasma-optical emission spectroscopy. To calculate removal efficiency (*RE*) and adsorption capacity (*q*_*e*_), the following equations (Eq. 8 and 9) were used:

8 $$RE \left(\%\right)=\frac{{C}_{i}-{C}_{e}}{{C}_{i}}\times 100$$

9 $${q}_{e}=\left(\frac{{C}_{i}-{C}_{e}}{W}\right)\times V$$

where *C*_*i*_, *C*_*e*_, *V*, and *W* are the initial concentrations of Pb(II) in the solution (mg L^−1^), the equilibrium concentrations of Pb(II) in the solution (mg L^-1^), the solution volume (mL), and the adsorbent amount (mg), respectively.

## References

[1] Davis ME (2002). Ordered porous materials for emerging applications. Nature.

[2] Taguchi A, Schüth F (2005). Ordered mesoporous materials in catalysis. Microporous Mesoporous Mater..

[3] Wu Z, Zhao D (2011). Ordered mesoporous materials as adsorbents. Chem. Commun..

[4] Hartmann M (2005). Ordered mesoporous materials for bioadsorption and biocatalysis. Chem. Mater..

[5] Shahvar A, Soltani R, Saraji M, Dinari M, Alijani S (2018). Covalent triazine-based framework for micro solid-phase extraction of parabens. J. Chromatogr. A.

[6] Saraji M, Shahvar A (2017). Metal-organic aerogel as a coating for solid-phase microextraction. Anal. Chim. Acta.

[7] Saraji M, Shahvar A (2016). Selective micro solid-phase extraction of epinephrine, norepinephrine and dopamine from human urine and plasma using aminophenylboronic acid covalently immobilized on magnetic nanoparticles followed by high-performance liquid chromatography-fluorescence detection. Anal. Methods.

[8] Modheji, M., Emadi, H. & Vojoudi, H. Efficient pre-concentration of As (III) in food samples using guanidine-modified magnetic mesoporous silica. *J. Porous Mater.* 1–8 (2020).

[9] Vojoudi H (2017). Post-modification of nanoporous silica type SBA-15 by bis (3-triethoxysilylpropyl) tetrasulfide as an efficient adsorbent for arsenic removal. Powder Technol..

[10] Banaei A (2018). 2, 2’-(butane-1, 4-diylbis (oxy)) dibenzaldehyde cross-linked magnetic chitosan nanoparticles as a new adsorbent for the removal of Reactive red 239 from aqueous solutions. Mater. Chem. Phys..

[11] Vojoudi H, Bastan B, Ghasemi JB, Badiei A (2019). An ultrasensitive fluorescence sensor for determination of trace levels of copper in blood samples. Anal. Bioanal. Chem..

[12] Aghaei, Z. *et al.* Sulfidic GO-grafted glass stir-bar as a noble metal ions adsorbent. *Microchem. J.* 104878 (2020).

[13] Moja T (2020). Melt processing of polypropylene-grafted-maleic anhydride/Chitosan polymer blend functionalized with montmorillonite for the removal of lead ions from aqueous solutions. Sci. Rep..

[14] Kurniawan TA (2021). Resource recovery toward sustainability through nutrient removal from landfill leachate. J. Environ. Manag..

[15] Kurniawan TA (2021). A societal transition of MSW management in Xiamen (China) toward a circular economy through integrated waste recycling and technological digitization. Environ. Pollut..

[16] Soltani R (2021). Novel bimodal micro-mesoporous Ni50Co50-LDH/UiO-66-NH2 nanocomposite for Tl(I) adsorption. Arab. J. Chem..

[17] Pelalak R (2021). Oak wood ash/GO/Fe3O4 adsorption efficiencies for cadmium and lead removal from aqueous solution: Kinetics, equilibrium and thermodynamic evaluation. Arab. J. Chem..

[18] Borghei SA (2021). Synthesis of multi-application activated carbon from oak seeds by KOH activation for methylene blue adsorption and electrochemical supercapacitor electrode. Arab. J. Chem..

[19] Pelalak R (2021). Molecular dynamics simulation of novel diamino-functionalized hollow mesosilica spheres for adsorption of dyes from synthetic wastewater. J. Mol. Liq..

[20] Marjani A, Zare MH, Sadeghi MH, Shirazian S, Ghadiri M (2021). Synthesis of alginate-coated magnetic nanocatalyst containing high-performance integrated enzyme for phenol removal. J. Environ. Chem. Eng..

[21] Soltani R, Marjani A, Shirazian S (2020). A hierarchical LDH/MOF nanocomposite: Single, simultaneous and consecutive adsorption of a reactive dye and Cr(vi). Dalton Trans..

[22] Shirazian S, Ashrafizadeh SN (2015). LTA and ion-exchanged LTA zeolite membranes for dehydration of natural gas. J. Ind. Eng. Chem..

[23] Soltani R, Marjani A, Hosseini M, Shirazian S (2020). Meso-architectured siliceous hollow quasi-capsule. J. Colloid Interface Sci..

[24] Soltani R, Madani A, Hosseini M, Shirazian S (2020). Mesostructured hollow siliceous spheres for adsorption of dyes. Chem. Eng. Technol..

[25] Soltani, R., Marjani, A., Moguei, M. R. S., Rostami, B. & Shirazian, S. Novel diamino-functionalized fibrous silica submicro-spheres with a bimodal-micro-mesoporous network: Ultrasonic-assisted fabrication, characterization, and their application for superior uptake of Congo red. *J. Mol. Liq.* **294**, 10.1016/j.molliq.2019.111617 (2019).

[26] Soltani, R., Marjani, A. & Shirazian, S. Shell-in-shell monodispersed triamine-functionalized SiO2 hollow microspheres with micro-mesostructured shells for highly efficient removal of heavy metals from aqueous solutions. *J. Environ. Chem. Eng.* **7**, 10.1016/j.jece.2018.102832 (2019).

[27] Soltani, R., Marjani, A., Hosseini, M. & Shirazian, S. Synthesis and characterization of novel N-methylimidazolium-functionalized KCC-1: A highly efficient anion exchanger of hexavalent chromium. *Chemosphere* **239**, 10.1016/j.chemosphere.2019.124735 (2020). 10.1016/j.chemosphere.2019.124735 31499306

[28] Shirazian S, Ashrafizadeh SN (2015). Synthesis of substrate-modified LTA zeolite membranes for dehydration of natural gas. Fuel.

[29] Cao Y (2021). Molecular dynamic simulations and quantum chemical calculations of adsorption process using amino-functionalized silica. J. Mol. Liq..

[30] Soltani R, Pelalak R, Pishnamazi M, Marjani A, Shirazian S (2021). A water-stable functionalized NiCo-LDH/MOF nanocomposite: Green synthesis, characterization, and its environmental application for heavy metals adsorption. Arab. J. Chem..

[31] Heidari Z (2021). Molecular modeling investigation on mechanism of cationic dyes removal from aqueous solutions by mesoporous materials. J. Mol. Liq..

[32] Liu, C., Yuan, P. & Cui, C. The pore confinement effect of FDU-12 mesochannels on MoS2 active phases and their hydrodesulfurization performance. *J. Nanomater.* **2016** (2016).

[33] Wu Q (2018). Synthesis and characterization of beta-FDU-12 and the hydrodesulfurization performance of FCC gasoline and diesel. Fuel Process. Technol..

[34] Fan J (2003). Cubic mesoporous silica with large controllable entrance sizes and advanced adsorption properties. Angew. Chem. Int. Ed..

[35] Fan J (2005). Low-temperature strategy to synthesize highly ordered mesoporous silicas with very large pores. J. Am. Chem. Soc..

[36] Yu T (2006). Pore structures of ordered large cage-type mesoporous silica FDU-12s. J. Phys. Chem. B.

[37] Soltani R, Dinari M, Mohammadnezhad G (2018). Ultrasonic-assisted synthesis of novel nanocomposite of poly (vinyl alcohol) and amino-modified MCM-41: A green adsorbent for Cd (II) removal. Ultrason. Sonochem..

[38] Mohammadnezhad G, Dinari M, Soltani R, Bozorgmehr Z (2015). Thermal and mechanical properties of novel nanocomposites from modified ordered mesoporous carbon FDU-15 and poly (methyl methacrylate). Appl. Surf. Sci..

[39] Morales-Luckie, R. A. *et al.* Facile solventless synthesis of a nylon-6, 6/silver nanoparticles composite and its XPS study. *Int. J. Polymer Sci.* **2013** (2013).

[40] Wei L, Hu N, Zhang Y (2010). Synthesis of polymer—Mesoporous silica nanocomposites. Materials.

[41] Müller K (2017). Review on the processing and properties of polymer nanocomposites and nanocoatings and their applications in the packaging, automotive and solar energy fields. Nanomaterials.

[42] Albadarin AB (2017). Activated lignin-chitosan extruded blends for efficient adsorption of methylene blue. Chem. Eng. J..

[43] Marjani, A., Soltani, R., Pishnamazi, M., Rezakazemi, M. & Shirazian, S. Functionalized pollen-like mesoporous silica. *Microporous Mesoporous Mater. ***310**, 10.1016/j.micromeso.2020.110531 (2021).

[44] Roozbeh S, Marjani A, Shirazian S (2020). A hierarchical LDH/MOF nanocomposite: Single, simultaneous and consecutive adsorption of a reactive dye and Cr (vi). Dalton Trans..

[45] Soltani R, Marjani A, Soltani R, Shirazian S (2020). Hierarchical multi-shell hollow micro–meso–macroporous silica for Cr (Vi) adsorption. Sci. Rep..

[46] Soltani, R. *et al.* Preparation of COOH-KCC-1/polyamide 6 composite by in situ ring-opening polymerization: Synthesis, characterization, and Cd(II) adsorption study. *J. Environ. Chem. Eng.* 104683, 10.1016/j.jece.2020.104683 (2020).

[47] Carmona D, Balas F, Santamaria J (2014). Pore ordering and surface properties of FDU-12 and SBA-15 mesoporous materials and their relation to drug loading and release in aqueous environments. Mater. Res. Bull..

[48] Kao H-M, Chang P-C, Wu J-D, Chiang AS, Lee C-H (2006). Direct synthesis, characterization and solid-state NMR spectroscopy of large-pore vinyl-functionalized cubic mesoporous silica FDU-12. Microporous Mesoporous Mater..

[49] Sarvi MN (2014). Development of functionalized mesoporous silica for adsorption and separation of dairy proteins. Chem. Eng. J..

[50] Deka JR (2015). Roles of nanostructures and carboxylic acid functionalization of ordered cubic mesoporous silicas in lysozyme immobilization. Microporous Mesoporous Mater..

[51] Hodgkins RP, Garcia-Bennett AE, Wright PA (2005). Structure and morphology of propylthiol-functionalised mesoporous silicas templated by non-ionic triblock copolymers. Microporous Mesoporous Mater..

[52] Cui H-Z (2017). Novel Pb (II) ion-imprinted materials based on bis-pyrazolyl functionalized mesoporous silica for the selective removal of Pb (II) in water samples. Microporous Mesoporous Mater..

[53] Liu, Q. & Dong, H. In-situ immobilizing Ni nanoparticles to FDU-12 via trehalose with fine size and location control for CO2 methanation. *ACS Sustain. Chem. Eng.* (2020).

[54] Huang L, Yan X, Kruk M (2010). Synthesis of ultralarge-pore FDU-12 silica with face-centered cubic structure. Langmuir.

[55] Meng Q (2018). Synthesis of zirconium modified FDU-12 by different methods and its application in dibenzothiophene hydrodesulfurization. RSC Adv..

[56] Mohammadnezhad G, Abad S, Soltani R, Dinari M (2017). Study on thermal, mechanical and adsorption properties of amine-functionalized MCM-41/PMMA and MCM-41/PS nanocomposites prepared by ultrasonic irradiation. Ultrason. Sonochem..

[57] Charles J, Ramkumaar G, Azhagiri S, Gunasekaran S (2009). FTIR and thermal studies on nylon-66 and 30% glass fibre reinforced nylon-66. J. Chem..

[58] Ghadami Jadval Ghadam, A. & Karimi, H. Synthesis and characterization of polyamide-66/calcium carbonate composites. *J. Chem. Petrol. Eng.* **49**, 63–78 (2015).

[59] Lawrence G, Anand C, Strounina E, Vinu A (2014). Biomolecule encapsulation over mesoporous silica with ultra-large tuneable porous structure prepared by high temperature microwave process. Sci. Adv. Mater..

[60] Nyairo WN (2018). Efficient adsorption of lead (II) and copper (II) from aqueous phase using oxidized multiwalled carbon nanotubes/polypyrrole composite. Sep. Sci. Technol..

[61] Liu Y (2011). Selective adsorption behavior of Pb (II) by mesoporous silica SBA-15-supported Pb (II)-imprinted polymer based on surface molecularly imprinting technique. J. Hazard. Mater..

[62] Zulfiqar M (2020). Efficient removal of Pb (II) from aqueous solutions by using oil palm bio-waste/MWCNTs reinforced PVA hydrogel composites: kinetic, isotherm and thermodynamic modeling. Polymers.

[63] Zarei F, Marjani A, Soltani R (2019). Novel and green nanocomposite-based adsorbents from functionalised mesoporous KCC-1 and chitosan-oleic acid for adsorption of Pb (II). Eur. Polymer J..

[64] Badruddoza AZM, Shawon ZBZ, Tay WJD, Hidajat K, Uddin MS (2013). Fe3O4/cyclodextrin polymer nanocomposites for selective heavy metals removal from industrial wastewater. Carbohyd. Polym..

